# Propensity-score-matching analysis to compare efficacy and safety between 16-gauge and 18-gauge needle in ultrasound-guided biopsy for peripheral pulmonary lesions

**DOI:** 10.1186/s12885-021-08126-7

**Published:** 2021-04-09

**Authors:** Weijun Huang, Jieyi Ye, Yide Qiu, Weiwei Peng, Ninghui Lan, Weizhen Cui, Ting Huang, Yinghui Ou, Yingjia Li

**Affiliations:** 1 Department of Medical Ultrasonics, Nanfang Hospital, Southern Medical University, No. 1838, North Guangzhou Avenue, Guangzhou, 510515 Guangdong China; 2 Division of Interventional Ultrasound, Department of Medical Ultrasonics, Foshan First People’s Hospital (The Affiliated Foshan Hospital of Sun Yat-sen University), 81 Lingnan North Road, Foshan, 528000 Guangdong China

**Keywords:** Efficacy, Safety, Ultrasound-guided biopsy, Peripheral pulmonary lesion, Propensity score matching analysis

## Abstract

**Background:**

Definitive diagnosis of peripheral pulmonary lesions (PPLs) depends on the histological analysis of the pleural biopsy sample. Ultrasound (US)-guided sampling is now standard practice in the clinical setting. However, determining a suitable needle size and sampling times to improve the efficacy and safety of the biopsy remains challenging. Here, we compared the efficacy between 16- and 18-gauge core biopsy needles in US-guided percutaneous transthoracic biopsy for PPLs on histological diagnosis and procedure-related complications.

**Materials and methods:**

In total, 1169 patients (767 men, 402 women; mean age, 59.4 ± 13.2 years) who received biopsy for PPLs between September 2011 and February 2019 were included. The propensity score matching (PSM) analysis was performed to adjust the baseline differences, and the rate of successful specimen assessment and complications were compared between the 16-gauge (249 patients) and 18-gauge (920 patients) groups. The number of pleural surfaces crossed (NOPSC) was defined as the number of times the visceral pleural surface was transgressed. Stratified analysis was performed based on NOPSC.

**Results:**

The overall success rate was 92.0% (1076/1169). The overall complication rate was 9.6%, including pneumothorax, hemorrhage, and vasovagal reaction, which occurred in 2.5% (29/1169), 6.6% (77/1169), and 0.5% (6/1169) of the patients, respectively. When NOPSC was 1 or > 2, the success and complication rates in the 16-gauge group were comparable to those of the 18-gauge group (all *P* > 0.05). When the NOPSC was 2, the success rate in the 16-gauge group was significantly higher than that in the 18-gauge group (*P* = 0.017), whereas the complication rate was comparable (*P* > 0.05).

**Conclusion:**

Higher success rate could be achieved using a 16-gauge than an 18-gauge core biopsy needle in the US-guided percutaneous transthoracic biopsy for PPLs when the NOPSC was 2. We recommend using 16-gauge needles with 2 times of needle passes in biopsy for PPLs in clinical practice.

## Background

The increasing importance of regular screening for lung cancer in recent times has made it possible to more commonly detect asymptomatic pulmonary nodules, including those located peripherally. However, diagnosing peripheral pulmonary lesions (PPLs), defined as nodules directly in contact with the chest wall without an intervening aerated lung [1], continues to be a challenge [2–4]. Surgical biopsy, bronchoscopy, and percutaneous biopsy are frequently used to diagnose PPLs [5]. Surgical biopsy is a classical approach to obtain an adequate amount of the diseased tissue for histopathological analysis, although it requires general anesthesia and is traumatic to the patient [6, 7]. On the contrary, bronchoscopy is relatively safe, but the diagnostic yield for PPLs is inadequate [8–10].

Percutaneous biopsy is more effective and less invasive than surgical biopsy [11, 12]. Computed tomography (CT)-guided percutaneous needle biopsy for PPLs is frequently used but is limited by radiation exposure and non-real-time monitoring [13]. In addition, the rate of post-procedure complications with percutaneous biopsy was reported to be high [14]. On the contrary, the real-time ultrasound (US)-guided percutaneous needle biopsy for PPLs is more beneficial in terms of being radiation-free, convenient, economical and dynamic than the CT-guided procedure [15–17]. Therefore, the US-guided biopsy for PPLs is considered a potentially feasible and reliable technique [18].

Certain studies have verified the efficacy and safety of US-guided biopsy for PPLs [16, 18, 19]. However, these procedures were commonly performed with an 18- or 20-gauge core needle. The needle size may be an important aspect, in addition to factors related to the patient, lesion, and procedure, contributing to a safe and successful biopsy, which requires further scrutiny for several reasons. First, biopsy specimens must provide enough tissue to guarantee both histological diagnosis and immunohistochemical analysis, and a larger needle helps obtain more specimens. Second, the use of a larger needle is more likely to cause complications, theoretically. Third, the choice of the needle size is an operator-controlled factor and can be easily changed. To the best of our knowledge, no large-sample studies have authenticated these assumptions. In addition to needle size, the concept of number of pleural surfaces crossed (NOPSC) was introduced in the study, which was defined as the number of times the visceral pleural surface was transgressed. A previous study demonstrated NOPSC was one of the factors associated with complications in CT-guided procedure [20]. Here, we compared the effect between the use of 16- and 18-gauge core needles in US-guided percutaneous biopsy for PPLs on histological diagnostic efficacy and procedure-related complications. To avoid possible biases and confounding factors, we applied the propensity score-matching (PSM) approach [21].

## Materials and methods

### Patients selection

All patients who received US-guided percutaneous transthoracic biopsy for PPLs with a 16- or 18-gauge core biopsy needle at our institution from September 2011 to February 2019 were included in the study. Patients with PPLs clearly displayed on ultrasonography and able to tolerate procedural positions and respond to breathing instructions were included. Patients with the following conditions were excluded: (a) pleural effusion, (b) biopsy intolerance due to severe cough or cardiopulmonary dysfunction, and (c) abnormal platelet number or prolonged blood clotting time. In the case of multiple PPLs, the most clearly seen lesion with a safe puncture path on the ultrasonography was selected as the main tumor for biopsy.

### Data collection

This retrospective study was approved by the institutional review board of Foshan First People’s Hospital. Informed consent was obtained from all participants. All procedures were performed in accordance with institutional and national standards on human experimentation, which complied with the Declaration of Helsinki of 1964 and its later amendments. The following data were obtained: demographic information (e.g., age, gender), patient position during the procedure, presence of emphysema, location and diameter of the lesion, and proportion of necrosis in the lesion. The pre-procedural contrast-enhanced CT (CECT) was evaluated by 2 radiologists, each with nearly 5 years of experience in using the technique. Any disagreement was resolved by discussion until consensus was reached. The diameter of the lesion was determined by the long-axis measurement in the axial plane on CT. The proportion of necrosis in the lesions, which manifested as non-enhanced areas on CECT, was recorded and classified into 2 groups (< 50% and ≥ 50%).

### US-guided percutaneous needle biopsy

**Fig. 1:**
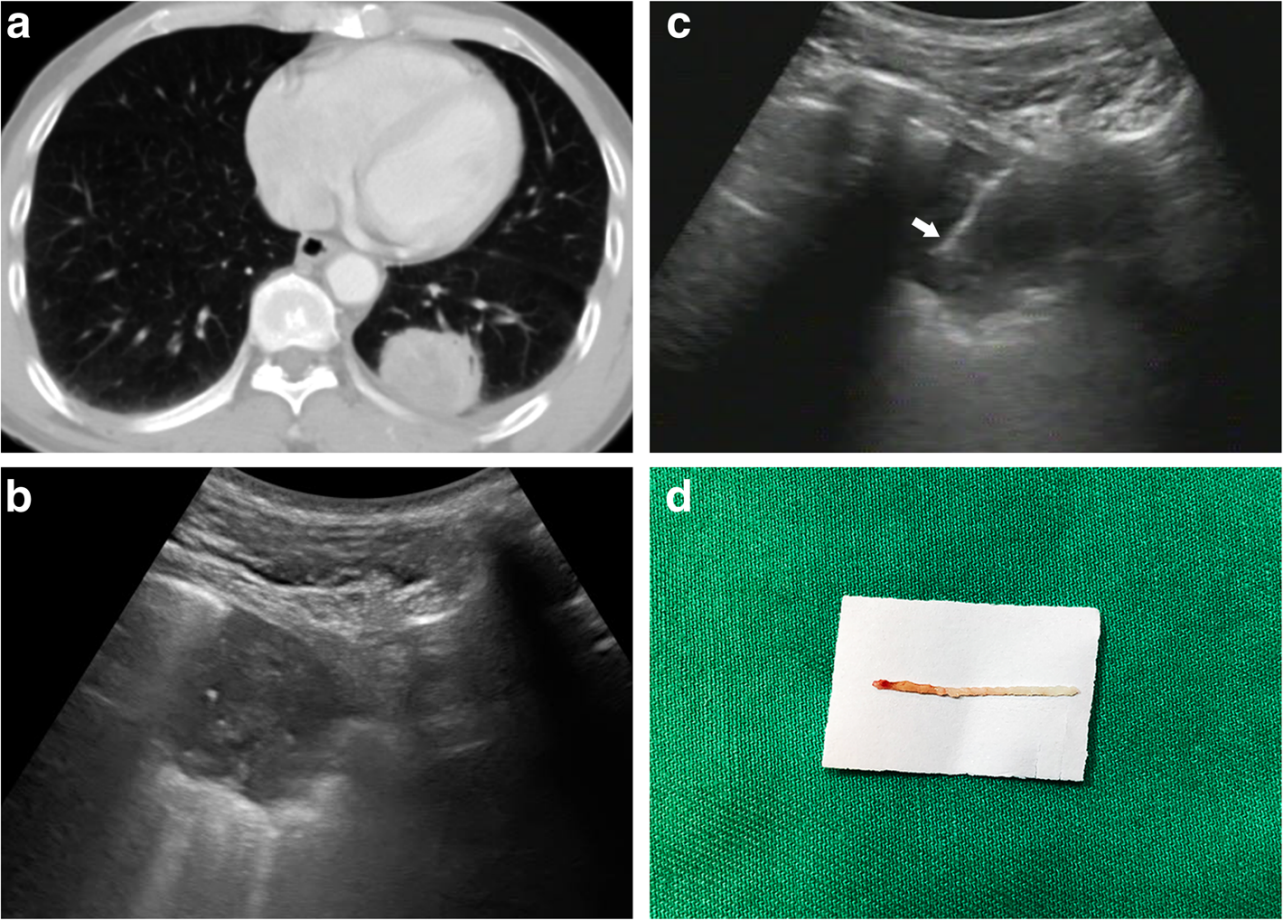
A 57-year-old patient with a peripheral lung lesion. A percutaneous core biopsy was requested to determine the nature of the lesion. **a** Axial contrast-enhanced computed tomography (CT) image revealing a round lung lesion in the left lower lobe with pleural contact. **b** Transverse gray-scale ultrasound (US) image obtained before biopsy revealing a hypoechoic lesion relative to the surrounding aerated lung. Positioning a transducer in the adjacent rib space shows broad pleural contact, providing a sonographic window for sampling. **c** Transverse gray-scale US image revealing a 16-gauge needle throw for core biopsy within the lesion (arrow), performed within a single breath-hold. **d** Color photograph of the biopsy sample

All patients received CECT examinations prior to the procedure to confirm the lesion location and to obtain a feasible sonographic window before the biopsy. The lesion location and characteristics were assessed on pre-biopsy CT images, and a suitable patient positioning was determined. Patient position (supine, prone, or lateral) was decided based on the location of the PPL and planned puncture pathway. The US-guided biopsies were performed by an interventional physician with 5 years of experience. We used a MyLab Twice machine (Esoate, Genoa, Italy) equipped with a convex array probe CA541 (frequency range: 1–8 MHz) for ultrasonography. The biopsy was performed with a core needle (Fig. 1). The adjustable biopsy gun (MG1522, BARD Magnum, BARD Peripheral Vascular, Tempe, AZ, USA) equipped with optional penetration depths of 15 or 22 mm for drawing out the specimen, and supplementary 18- or 16-gauge biopsy needles (BARD Magnum Disposable Needle, BARD Peripheral Vascular), were used in all procedures. All needles were 20 cm in length, and the diameters of biopsy notch in 16- and 18-gauge needles were 1.6 and 1.2 mm, respectively. An 18-gauge needle was used for procedures performed between September 2011 and July 2017, and a 16-gauge needle was used up to February 2019.

Local anesthesia was administered to the disinfected biopsy site. Real-time color Doppler imaging was used to avoid vessels. The freehand technique was employed in all cases. The needle was introduced and gently advanced toward the lesion of interest. When the needle was advanced in the lesion, the biopsy was performed, and the needle was then removed. The penetration depth was 22 mm for drawing out the specimens in all patients. Biopsies were performed during patient breath-holding. An enhanced solid portion of the lesion on CECT was assumed to be the biopsy target area, while non-enhancing areas were avoided as far as possible. Samples were assessed visually for adequacy. If the specimen was highly fragmented for histological examination, a repeat biopsy was conducted until adequate tissues were obtained. Biopsies were performed 1–4 times as tolerated by the patient. NOPSC was defined as the number of times the visceral pleural surface was transgressed. Kuban et al. [20] in their study on CT-guided biopsy for lung lesions, first proposed the NOPSC. For example, if a fissure is crossed during the CT-guided biopsy, the NOPSC is 3. In the US-guided biopsy for PPLs, the NOPSC refers to the number of biopsies because only the visceral pleural outside the lesion would be transgressed in one biopsy section. The sample was conserved in a formalin container and delivered to histopathological analysis.

### Complication evaluations

Post-procedure, patients were shifted to the ward and instructed to avoid getting out of the bed for at least 6 h. The patient’s vital signs and symptoms, hemoglobin levels, and imaging were closely observed for at least 24 h. Chest radiographs and ultrasonography were performed 1 h after the procedure to detect complications, such as pneumothorax and hemothorax, and if needed, further follow-up radiographs were performed [22, 23]. The vasovagal reaction is common during the procedure, manifesting as lightheadedness, hypotension, nausea, and/or transient bradycardia [24, 25].

The criterion of a large pneumothorax is the existence of a visible rim of > 2 cm between the lung margin and inner chest wall, which can be measured on a chest radiograph at the level of the hilum [26]. In our study, the severity of hemorrhage was classified as minor and major based on the fluid-free area in the ultrasonography. Minor hemorrhage was defined as hemorrhage < 2 cm in width or only hemoptysis that did not cause obvious shortness of breath and blood oxygen saturation reduction, whereas major hemorrhage was defined as hemorrhage > 2 cm. Chest tube placement was based on the severity of symptoms and/or degree of lung compression. The tube was removed when the symptom, vital signs, and chest radiograph demonstrated that the complication was resolved. A repeat chest radiograph was performed prior to discharge.

### Pathological evaluations

Biopsy samples were routinely embedded in paraffin. Thin sections were cut and stained with hematoxylin and eosin. Immunohistochemical analysis was performed if needed. Two pathologists reviewed microscopic sections, each with about 10 years of experience in pulmonary pathology, and a final diagnosis was determined through consensus.

Based on the histological findings of the biopsy samples, lesions were categorized into 3 groups: malignant, benign, and non-diagnostic groups. The malignant group included lesions with a finding of any malignant diseases. The benign group included lesions with definite benign features, such as tuberculoma, organizing pneumonia, chronic inflammation, granulomatous inflammation, abscess, pulmonary mycosis, or other benign tumors. The non-diagnostic group included biopsy specimens deemed insufficient for diagnosis or showed few atypical cells. The malignant and benign groups reflected successful biopsies.

### Statistical analysis

The statistical analysis was performed using SPSS 22.0 (SPSS Inc., Chicago, IL, USA). Chi-square or Fisher exact test was applied to compare the difference of the categorical variable. Quantitative data are expressed as mean ± standard deviation. The difference of the quantitative variable was determined using independent sample T or Mann-Whitney U test. A *P* value of < 0.05 indicated a statistically significant difference. To reduce the bias from baseline confounding variables, the PSM analysis was performed to recognize a cohort of participants with similar baseline characteristics (Tables 2, 3 and 4).

**Fig. 2:**
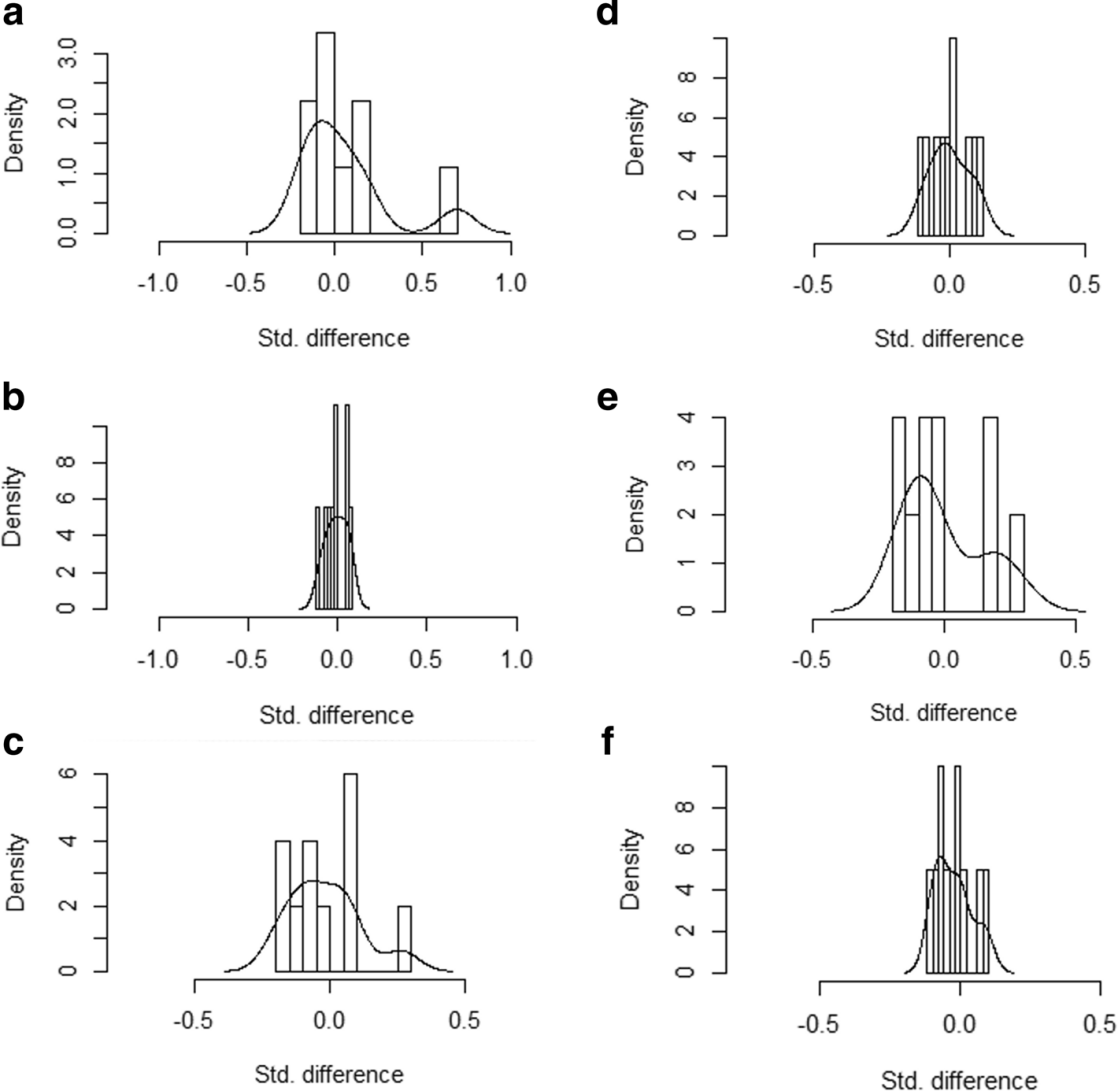
Histograms of standardized differences before and after propensity score-matching analysis. **a** A histogram of standardized differences before the propensity score-matching (PSM) analysis when the number of pleural surfaces crossed (NOPSC) was 1. **b** A histogram of standardized differences after the PSM analysis when NOPSC was 1. The standardized differences after matching are concentrated around 0, indicating good matching performance. **c** A histogram of standardized differences before the PSM analysis when NOPSC was 2. **d** A histogram of standardized differences after the PSM analysis when NOPSC was 2. The standardized differences after matching are concentrated around 0, indicating good matching performance. **e** A histogram of standardized differences before the PSM analysis when NOPSC was 3. **f** A histogram of standardized differences after the PSM analysis when NOPSC was 3. The standardized differences after matching are concentrated around 0, indicating good matching performance

The propensity score is a conditional probability of having a particular exposure (16- vs. 18-gauge needle) given a set of measured covariates at baseline. The propensity score was evaluated using a non-parsimonious multivariable logistic regression model. The 16-gauge group served as the dependent variable, and statistically significant baseline characteristics, including age (years), gender (male or female), patient position (supine, prone, or lateral), emphysema on CT, location of PPLs (left upper lobe, left lower lobe, right upper lobe, right middle lobe, or right lower lobe), the diameter of the lesion (cm), the proportion of necrosis in the lesion (< 50% or ≥ 50%), served as covariates. The PSM was performed with a 1:1 matching protocol without replacement (greedy-matching algorithm). The caliper width was equal to 0.2 of the standard deviation of the logit of the propensity score. After the PSM, the baseline characteristics were compared between the groups to re-evaluate the comparability. Histograms of standardized differences before and after propensity score-matching analysis were plotted to evaluate the matching performance intuitively (Fig. 2).

## Results

### Patients and lesion profile

A total of 1169 patients were enrolled in this study. The baseline characteristics and lesion profiles are summarized in Table 1. There were 767 (65.6%) men and 402 (34.4%) women, with a mean age of 59.4 ± 13.2 years (range, 18–85 years). Of these patients, 249 (21.3%) and 920 (78.7%) received biopsies with 16- and 18-gauge needles, respectively. The average diameter of the lesions was 5.1 ± 2.6 cm (range, 0.7–11.6 cm). Overall, there were no significant differences between the 16-gauge (*n* = 249) and 18-gauge (*n* = 920) groups in terms of gender (*P* = 0.268), patient position (*P* = 0.719), PPL location (*P* = 0.590), and necrosis proportion in the lesion (*P* = 0.184). However, mean age (*P* = 0.028) and mean lesion diameter (*P* = 0.024) were significantly higher in the 16-gauge group. Moreover, there was a significant difference in the NOPSC (*P* < 0.001) and emphysema on CT (*P* = 0.044) between the groups.

**Table 1:** Characteristics and lesion profile of patients undergoing ultrasound-guided lung biopsy using 16-gauge and 18-gauge biopsy needle

Characteristics	16-gauge (*n* = 249)	18-gauge (*n* = 920)	*P* value
Age (years)	57.8 ± 14.5	60.0 ± 12.8	0.028
Gender			
Male/female	156/93	611/309	0.268
Patient position (%)			
Supine	93 (37.3%)	329 (35.8%)	0.719
Prone	112 (45.0%)	440 (47.8%)	
Lateral	44 (17.7%)	151 (16.4%)	
Emphysema on CT	19 (7.6%)	112 (12.2%)	0.044
Location of pulmonary lesion (%)			
Left upper lobe	42 (16.9%)	193 (21.0%)	0.590
Left lower lobe	64 (25.7%)	207 (22.5%)	
Right upper lobe	64 (25.7%)	245 (26.6%)	
Right middle lobe	21 (8.4%)	75 (8.2%)	
Right lower lobe	58 (23.3%)	200 (21.7%)	
Diameter of lesion (cm)	4.8 ± 2.5	5.2 ± 2.6	0.024
< 5	119 (47.8%)	439 (47.7%)	0.984
≥ 5	130 (52.2%)	481 (52.3%)	
Proportion of necrosis in the lesion			
< 50%	215 (86.3%)	762 (82.8%)	0.184
≥ 50%	34 (13.7%)	158 (17.2%)	
Number of pleural surfaces crossed			
1	37 (14.9%)	56 (6.1%)	< 0.001
2	147 (59.0%)	647 (70.3%)	
> 2	65 (26.1%)	217 (23.6%)	
Success rate (%)	240 (96.4%)	836 (90.9%)	0.004
Malignant	135 (54.2%)	543 (59.1%)	0.001
Benign	105 (42.2%)	293 (31.8%)	
Non-diagnostic	9 (3.6%)	84 (9.1%)	
Complication rate (%)	24 (9.6%)	88 (9.6%)	0.972
Pneumothorax	4 (1.6%)	25 (2.7%)	0.317
Hemorrhage	15 (6.0%)	62 (6.8%)	0.687
Vasovagal reaction	5 (2.0%)	1 (0.1%)	0.002

In 93 patients with an NOPSC of 1, there were no significant differences between the 16-guage (*n* = 37) and 18-gauge (*n* = 56) groups in terms of age (*P* = 0.975), gender (*P* = 0.400), patient position (*P* = 0.780), emphysema on CT (*P* = 0.254), PPL location (*P* = 0.731), PPL diameter (*P* = 0.447), and necrosis proportion in the lesion (*P* = 1.000). After the PSM, a total of 36 patient pairs (1:1) were extracted, with no significant differences in baseline characteristics between the patients in these groups (all *P* > 0.05; Table 2).

**Table 2:** Comparison of characteristics, efficacy and safety in patients undergoing ultrasound-guided lung biopsy between 16-gauge and 18-gauge core biopsy needle when number of pleural surface crossed was 1

Variable	Overall cohort			Propensity-score matched cohort		
	16-gauge (*n* = 37)	18-gauge (*n* = 56)	*P* value	16-gauge (*n* = 36)	18-gauge (*n* = 36)	*P* value
Age (years)	59.0 ± 13.8	59.0 ± 13.3	0.975	58.5 ± 13.7	59.2 ± 12.6	0.823
Gender						
Male/female	25/12	33/23	0.400	24/12	21/15	0.465
Patient position						
Supine/prone/lateral	16/13/8	27/20/9	0.780	16/7/13	14/8/14	0.888
Emphysema on CT	0 (0%)	4 (7.1%)	0.254	0 (0%)	0 (0%)	–
Location of pulmonary lesion (%)						
Left upper lobe	10 (27.1%)	16 (28.6%)	0.731	9 (25.0%)	11 (30.6%)	0.674
Left lower lobe	9 (24.3%)	10 (17.9%)		9 (25.0%)	6 (16.7%)	
Right upper lobe	7 (18.9%)	14 (25.0%)		7 (19.4%)	7 (19.4%)	
Right middle lobe	2 (5.4%)	6 (10.6%)		2 (5.6%)	5 (13.9%)	
Right lower lobe	9 (24.3%)	10 (17.9%)		9 (25.0%)	7 (19.4%)	
Diameter of lesion (cm)	4.3 ± 2.2	4.7 ± 2.4	0.447	4.3 ± 2.3	4.4 ± 2.4	0.841
< 5/≥5	21/16	26/30	0.330	20/16	18/18	0.637
Proportion of necrosis in the lesion						
< 50%/≥50%	35/2	52/4	1.000	34/2	35/1	1.000
Success rate (%)	36 (97.3%)	54 (96.4%)	1.000	35 (97.2%)	34 (94.4%)	1.000
Malignant	23 (62.2%)	28 (50.0%)	0.514	13 (36.1%)	13 (36.1%)	0.837
Benign	13 (35.1%)	26 (46.4%)		22 (61.1%)	21 (58.3%)	
Non-diagnostic	1 (2.7%)	2 (3.6%)		1 (2.8%)	2 (5.6%)	
Complication rate (%)	1 (2.7%)	8 (14.3%)	0.081	1 (2.8%)	6 (16.7%)	0.112
Pneumothorax	0 (0%)	3 (5.4%)	0.406	0 (0%)	2 (5.6%)	0.473
Major	0 (0%)	0 (0%)	–	0 (0%)	0 (0%)	–
Minor	0 (0%)	3 (5.4%)	0.406	0 (0%)	2 (5.6%)	0.473
Hemorrhage	1 (2.7%)	5 (8.9%)	0.397	1 (2.8%)	4 (11.1%)	0.354
Major	0 (0%)	0 (0%)	–	0 (0%)	0 (0%)	–
Minor	1 (2.7%)	5 (8.9%)	0.397	1 (2.8%)	4 (11.1%)	0.354
Vasovagal reaction	0 (0%)	0 (0%)	–	0 (0%)	0 (0%)	–

In 749 patients with an NOPSC of 2, there were significant differences between the 16-guage (*n* = 147) and 18-gauge (*n* = 647) groups in terms of age (*P* = 0.016) and PPL diameter (*P* = 0.025). There were no significant differences between the groups in terms of gender (*P* = 0.424), patient position (*P* = 0.846), emphysema by CT (*P* = 0.308), PPL location (*P* = 0.831), and necrosis proportion in the lesion (*P* = 0.425). The PSM was subsequently performed to balance the difference between the groups. After matching, a total of 145 patient pairs (1:1) were extracted, with no significant differences in baseline characteristics between the patients in these groups (all *P* > 0.05; Table 3).

**Table 3:** Comparison of characteristics, efficacy and safety in patients undergoing ultrasound-guided lung biopsy between 16-gauge and 18-gauge core biopsy needle when number of pleural surface crossed was 2

Variable	Overall cohort			Propensity-score matched cohort		
	16-gauge (*n* = 147)	18-gauge (*n* = 647)	*P* value	16-gauge (*n* = 145)	18-gauge (*n* = 145)	*P* value
Age (years)	57.8 ± 14.2	60.6 ± 12.4	0.016	58.3 ± 13.6	58.1 ± 13.3	0.889
Gender						
Male/female	94/53	436/211	0.424	94/51	96/49	0.805
Patient position						
Supine/prone/lateral	52/69/26	232/313/102	0.846	51/69/25	48/74/23	0.840
Emphysema on CT	16 (10.9%)	91 (14.1%)	0.308	16 (11.0%)	18 (12.4%)	0.715
Location of pulmonary lesion (%)						
Left upper lobe	27 (18.4%)	128 (19.8%)	0.831	27 (18.7%)	31 (21.4%)	0.465
Left lower lobe	35 (23.8%)	144 (22.2%)		34 (23.4%)	42 (29.0%)	
Right upper lobe	34 (23.1%)	174 (26.9%)		34 (23.4%)	32 (22.0%)	
Right middle lobe	12 (8.2%)	51 (7.9%)		12 (8.3%)	14 (9.7%)	
Right lower lobe	39 (26.5%)	150 (23.2%)		38 (26.2%)	26 (17.9%)	
Diameter of lesion (cm)	4.8 ± 2.6	5.3 ± 2.6	0.025	4.8 ± 2.6	5.0 ± 2.4	0.582
< 5/≥5	70/77	313/334	0.868	68/77	69/76	0.906
Proportion of necrosis in the lesion						
< 50%/≥50%	123/24	523/124	0.425	121/24	113/32	0.234
Success rate (%)	141 (95.9%)	580 (89.6%)	0.017	139 (95.9%)	128 (88.3%)	0.017
Malignant	76 (51.7%)	393 (60.7%)	< 0.001	76 (52.4%)	89 (61.4%)	0.003
Benign	65 (44.2%)	187 (28.9%)		63 (43.5%)	39 (26.9%)	
Non-diagnostic	6 (4.1%)	67 (10.4%)		6 (4.1%)	17 (9.7%)	
Complication rate (%)	12 (8.2%)	56 (8.7%)	0.847	11 (7.6%)	10 (6.9%)	0.821
Pneumothorax	3 (2.0%)	14 (2.2%)	1.000	3 (2.1%)	4 (2.8%)	1.000
Major	2 (1.4%)	2 (0.3%)	0.158	2 (1.4%)	1 (0.7%)	1.000
Minor	1 (0.7%)	12 (1.9%)	0.514	1 (0.7%)	3 (2.1%)	0.615
Hemorrhage	8 (5.5%)	44 (6.8%)	0.209	7 (4.8%)	6 (4.1%)	0.777
Major	0 (0%)	0 (0%)	–	0 (0%)	0 (0%)	–
Minor	8 (5.5%)	44 (6.8%)	0.548	7 (4.8%)	6 (4.1%)	0.777
Vasovagal reaction	1 (0.7%)	0 (0%)	0.185	1 (0.7%)	0 (0%)	1.000

In 282 patients with an NOPSC of > 2, there were no significant differences between the 16- (*n* = 65) and 18-gauge (*n* = 217) groups in terms of age (*P* = 0.719), gender (*P* = 0.211), patient position (*P* = 0.626), emphysema by CT (*P* = 0.541), PPL location (*P* = 0.071), PPL diameter (*P* = 0.475), and necrosis proportion in the lesion (*P* = 0.753). After the PSM, a total of 63 patient pairs (1:1) were extracted, with no significant differences in baseline characteristics between the patients in these groups (all *P* > 0.05; Table 4).

**Table 4:** Comparison of characteristics, efficacy and safety in patients undergoing ultrasound-guided lung biopsy between 16-gauge and 18-gauge core biopsy needle when number of pleural surfaces crossed was more than 2

Variable	Overall cohort			Propensity-score matched cohort		
	16-gauge (*n* = 65)	18-gauge (*n* = 217)	*P* value	16-gauge (*n* = 63)	18-gauge (*n* = 63)	*P* value
Age (years)	57.1 ± 15.9	57.8 ± 13.7	0.719	57.4 ± 16.0	59.1 ± 12.7	0.514
Gender						
Male/female	37/28	142/75	0.211	37/26	35/28	0.719
Patient position						
Supine/prone/lateral	25/30/10	70/107/40	0.626	23/10/30	20/10/33	0.839
Emphysema on CT	3 (4.6%)	17 (7.8%)	0.541	3 (4.8%)	4 (6.3%)	1.000
Location of pulmonary lesion (%)						
Left upper lobe	5 (7.7%)	49 (22.6%)	0.071	5 (7.9%)	11 (17.5%)	0.251
Left lower lobe	20 (30.8%)	53 (24.4%)		20 (31.8%)	13 (20.6%)	
Right upper lobe	23 (35.3%)	57 (26.3%)		23 (36.5%)	18 (28.6%)	
Right middle lobe	7 (10.8%)	18 (8.3%)		6 (9.5%)	9 (14.3%)	
Right lower lobe	10 (15.4%)	40 (18.4%)		9 (14.3%)	12 (19.0%)	
Diameter of lesion (cm)	5.0 ± 2.3	5.2 ± 2.6	0.475	5.0 ± 2.3	5.0 ± 2.7	0.968
< 5/≥5	28/37	100/117	0.669	27/36	31/32	0.475
Proportion of necrosis in the lesion						
< 50%/≥50%	57/8	187/30	0.753	55/8	57/6	0.571
Success rate (%)	63 (96.9%)	202 (93.1%)	0.254	61 (96.8%)	59 (93.7%)	0.676
Malignant	36 (55.4%)	122 (56.2%)	0.469	35 (55.6%)	32 (50.8%)	0.664
Benign	27 (41.5%)	80 (36.9%)		26 (41.2%)	27 (42.9%)	
Non-diagnostic	2 (3.1%)	15 (6.9%)		2 (3.2%)	4 (6.3%)	
Complication rate (%)	11 (16.9%)	22 (10.1%)	0.135	11 (17.5%)	5 (7.9%)	0.108
Pneumothorax	1 (1.5%)	8 (3.7%)	0.690	1 (1.6%)	2 (3.2%)	1.000
Major	1 (1.5%)	2 (0.9%)	0.546	1 (1.6%)	1 (1.6%)	1.000
Minor	0 (0%)	6 (3.8%)	0.387	0 (0%)	1 (1.6%)	1.000
Hemorrhage	6 (9.2%)	13 (6.0%)	0.398	6 (9.5%)	3 (4.8%)	1.000
Major	0 (0%)	0 (0%)	–	0 (0%)	0 (0%)	–
Minor	6 (9.2%)	13 (6.0%)	0.398	6 (9.5%)	3 (4.8%)	0.489
Vasovagal reaction	4 (6.2%)	1 (0.4%)	0.012	4 (6.3%)	0 (0%)	0.127

### Efficacy of US-guided core needle biopsy

The overall biopsy success rate was 92.0% (1076/1169). Among all biopsies, 678 PPLs were categorized as malignant: 581 non-small cell lung cancers, 43 metastatic lung cancers, 31 small cell lung cancers, 11 malignant lymphomas, 9 malignant mesenchymal tumors, and 3 malignant solitary fibromas, and 398 PPLs were categorized as benign: 197 chronic non-specific inflammation, 104 tuberculoma, 37 organizing pneumonias, 18 chronic granulomatous inflammation, 13 abscesses, 17 pulmonary mycosis, 6 benign solitary fibrous tumors, 4 inflammatory pseudotumors, and 2 benign spindle cell tumors. The remaining 93 biopsy samples were categorized as non-diagnostic. The biopsy success rate in the 16-gauge group (96.4%, 240/249) was significantly higher than that in the 18-gauge group (90.9%, 836/920).

Based on the NOPSC (NOPSC = 1, 2, > 2), data were categorized into 3 groups. When the NOPSC was 2, the success rate was significantly higher in the 16-gauge group than in the 18-gauge group in both overall and PSM cohorts (both *P* = 0.017). When the NOPSC was 1 or > 2, the success rate was comparable between the 16- and 18-gauge groups in both overall and PSM cohorts (all *P >* 0.05).

### Complications of US-guided core needle biopsy

The overall post-procedure complication rate was 9.6% (112/1169). None of these incidents resulted in permanent severe sequelae or death. The rate of complications in the 16-gauge group (9.6%, 24/249) was comparable to that in the 18-gauge group (9.6%, 88/920; *P* = 0.972). In total, pneumothorax occurred in 29 out of 1169 patients (2.5%), including 22 cases of minor pneumothorax and 7 major pneumothorax cases. When minor pneumothorax was detected, air escaped from the pleural during needle removal. Patients were then monitored in a puncture-site-down position immediately and treated with nasal oxygen for at least 3 h. Major pneumothorax was observed in 7 patients (0.6%) who required post-procedural treatment via pigtail catheter insertion. There was no significant difference in the rate of pneumothorax between the 16- and 18-gauge groups (1.6% vs. 2.7%, *P* = 0.317).

The overall rate of hemorrhage was 6.6% (77/1169). No major hemorrhage occurred. All hemorrhages were reported as minor and were observed until stable without deterioration. Minor hemorrhage occurred with or without hemoptysis. Pleural effusion and hemoptysis were recorded in 16 and 61 patients, respectively. In this study, the treatment of hemorrhage included placing the patient in a puncture-site-down position with the bleeding lung placed downward to make the non-operated lung free air accessible and administration of tranexamic acid 500–1000 mg intravenously (non-bolus). There was no significant difference in hemorrhage rate between the 16- and 18-gauge groups (6.0% vs. 6.8%, *P* = 0.687).

The overall rate of vasovagal reaction was 0.5% (6/1169). The reactions were managed by stopping the procedure immediately, placing the patient in a recumbent position, and elevating the lower extremities. The vital sign and consciousness were monitored closely, and the recovery time was quick. The rate of vasovagal reaction was significantly higher in the 16-gauge group than in the 18-gauge group (2.0% vs. 0.1%, *P* = 0.002).

When the NOPSC was 1, 2, or > 2, the rates of pneumothorax, hemorrhage, and vasovagal reaction in the 16-gauge group were comparable to those in the 18-gauge group in the PSM cohort (all *P* > 0.05).

## Discussion

Determining the needle size to be used in a biopsy for PPLs is an important consideration for radiologists performing thoracic interventions. Selecting the safest and most effective needle should be a priority. In previous studies on US-guided percutaneous biopsy for PPLs, the procedures were mainly performed with an ≤18-gauge needle, but the insufficient tissue yield still remained a challenge. Moreover, how to determine needle size and NOPSC had not been explored thoroughly. This study confirmed the high efficacy and safety of US-guided percutaneous transthoracic biopsy for PPLs and revealed that US-guided biopsy had a higher success rate without increased complications by using a 16-gauge needle when the NOPSC was 2. The 16-gauge needles with two needle passes in biopsy for PPLs are recommended in the clinical setting.

In this study, US-guided biopsies appeared to be safe, with an overall complication rate of 9.6%. No death or severe sequela resulted from the procedures. These findings are acceptable compared with those reported in previous studies, in which 2.1–12.8% of the patients experienced complications [1, 27, 28]. However, Guo et al. reported an overall complication rate of 12.8% in 637 PPLs, wherein a 16-gauge needle was applied in 24% of the lesions and an 18-gauge needle applied in 76% of the lesions [27]. In the study by Guo et al., more needle passes may have contributed to the slightly higher rate than that in the current study. The median NOPSC reached > 3 in the study by Guo et al., whereas it was 2 in our study. Furthermore, Guo et al. reported the occurrence of hemorrhage and pneumothorax in 8.0 and 1.7% of the patients, respectively [27], which are similar to that in our study. Only 0.6% of our patients experienced post-procedural pneumothorax that required chest catheter insertion and recovered within 3–5 days. In this study, hemoptysis was self-limiting, which was relieved by reassuring and positioning the patient laterally with the biopsy side down. Hemorrhage in the absence of hemoptysis is usually minor and often asymptomatic, but the patient may present with confusion from hypoxia or shock in case of increased bleeding. In the case of severe bleeding, patients may receive bronchoscopy with a tamponade, a balloon catheter, coagulopathy, and coiling. The vasovagal reaction is a relatively rare complication caused by reflex vagal hyperfunction induced by pleural stimulation, and it often does not require atropine. Pereyra et al. reported the occurrence of vasovagal reactions in 27 of the 678 blind closed biopsy procedures for pleural biopsy, for an incidence of 4.1% [25]. In our study, the rate was only 0.6%, mainly because of rapid puncture across the pleura when performing a biopsy to reduce the period of provoking the pleura.

US-guided percutaneous biopsy has been achieving gradual acceptance in the clinical setting. Compared with CT-guided procedures, real-time US-guided approaches have the advantages of being convenient, economical, and radiation-free. Importantly, the approach had success rates of 81.8–93.4% in previous studies [1, 28, 29]. In our study, the success rate of diagnosis was 92.0%. According to the present study, the success rate was higher with a 16-gauge needle than with an 18-gauge needle when the NOPSC was 2. The trend toward improved pathologic sample success with a 16-gauge needle mainly resulted from directly visualizing the sampling of PPLs under real-time US guidance and using a larger gauge needle. It is not difficult to understand that the amount of tissue is more when a 16-gauge needle is applied. Repeatability and adequate amount of the sample are known to increase the success rate. However, a suitable NOPSC to reach adequacy threshold for successful diagnosis using a 16-gauge needle remains unclear. In this study, a 16-gauge core biopsy needle was employed in about 21% of the cases, yielding a larger tissue sample and improving the success rate of pathological evaluation when the NOPSC was 2. First, 16-gauge needles are provided with larger biopsy notch than 18-guage needles. When the penetration depth was the same, 16-gauge needles could obtain more tissue in one biopsy section to preferably meet the sample adequacy for pathological evaluation. Besides, 16-gauge needles show more advantageous controllability than 18-gauge ones as a result of their larger size, which enables the physician to control the needle direction and ensure the accuracy of puncture site. Therefore, to achieve higher efficacy, a 16-gauge needle with 2 times of needle passes should be recommended for biopsy instead of an 18-gauge needle, as more amount of the tissue can be obtained for pathological analysis.

In addition to efficacy benefits, the overall complication rate was analogous between the 16- and 18-gauge needles when the NOPSC was fixed. Many of the factors that have been previously proved to predispose to complications are related to the patient or lesion and cannot be changed [19, 30]. Needle size, however, is well within the operator’s control. It was thought that complications increased with larger needle size, which tended to injure a considerable portion of the lung parenchyma and lead to increased air leakage and bleeding. However, the results demonstrated that the rates of pneumothorax, hemorrhage, and vasovagal reaction were not higher in the 16- than in the 18-gauge group when the NOPSC was fixed. It suggested that 16-gauge needles are as safe as 18-gauge needles when the needle passes are the same, and it is not necessary to choose 18-gauge needles to avoid post-procedural complications. In the 16-gauge group, the success rates were comparable between the NOPSC = 2 and NOPSC > 2 groups (*P* = 0.723). However, compared with NOPSC > 2, although the complication rate was not significantly higher when NOPSC = 2 (16.9% vs. 8.2%, *P* = 0.059), there was an observed uptrend. Therefore, to reduce complication occurrence and increase the success rate, a 16-gauge core needle with 2 times of needle passes is recommended for routine use.

There are several limitations to our study. First, selection bias is inevitable in a retrospective study. Patients were not randomized to a 16-gauge or 18-gauge group, which may have resulted in a selection bias, although this effect was minimized by using a PSM analysis. Second, the data were obtained from a single center. The results in this study may not represent the experiences of other institutions. Moreover, other potential risk factors not included in the study could not be evaluated for confounding effects, and a comparison with CT-guided biopsy was not performed. Therefore, further validation of the results of this study is warranted.

## Conclusions

US-guided percutaneous transthoracic biopsy for PPLs is an effective and safe procedure. A higher success rate could be achieved by using a 16- than an 18-gauge core biopsy needle when the NOPSC was 2. We recommend using 16-gauge needles with 2 times of needle passes in biopsy for PPLs in clinical practice.

## Data Availability

All the data and materials supporting the conclusions were included in the main paper. The datasets used in the current study could be available from the corresponding author on request.

## Abbreviations

PPLs: Peripheral pulmonary lesions
PSM: Propensity score matching
NOPSC: Number of pleural surfaces crossed
CT: Computed tomography
US: Ultrasound
CECT: Contrast-enhanced computed tomography
